# Seasonal and circadian rhythms of clerodane diterpenes and glycosylated flavonoids in two varieties of *Casearia sylvestris* Sw. (Salicaceae)

**DOI:** 10.1016/j.heliyon.2024.e39488

**Published:** 2024-10-17

**Authors:** Paula C.P. Bueno, Gabriel S. Viana, Livia L. Thomaz, Daniela A. Chagas-Paula, Michael Hippler, Alberto J. Cavalheiro

**Affiliations:** a Institute of Chemistry, São Paulo State University, UNESP, Francisco Degni 55, 14800-900, Araraquara, SP, Brazil; b Institute of Chemistry, Federal University of Alfenas, UNIFAL, Gabriel Monteiro da Silva 700, 37130-001, Alfenas, MG, Brazil; c Leibniz Institute of Vegetable and Ornamental Crops, IGZ, Theodor-Echtermeyer-Weg 1, 14979, Großbeeren, Germany; d Institut für Biologie und Biotechnologie der Pflanzen (IBBP), Westfälische Wilhelms-Universität Münster, Schlossplatz 8, 48143, Münster, Germany

**Keywords:** *Casearia sylvestris* Sw., Salicaceae, Seasonal variation, Circadian rhythm, Clerodane diterpene, Glycosylated flavonoid

## Abstract

*Casearia sylvestris* Sw. (Salicaceae) is noted for its morphological and chemical plasticity and pharmacological properties. The present study investigates two of its varieties: *C. sylvestris* var. *sylvestris*, predominant in dense and humid forests and ecotones and characterized by clerodane diterpenes; and *C. sylvestris* var. *lingua*, mainly found in xeric and open savannah areas and containing phenolic compounds. Despite their comprehensive chemical profiles, the dynamics of clerodane diterpenes and glycosylated flavonoids remain unknown. This study thus aimed to describe seasonal and circadian variations in their content in the leaves of the two varieties. The relative contents of five diterpenes and three glycosylated flavonoids were monitored monthly, every 3 h for 48 h, over 1 year via high-performance liquid chromatography coupled to diode array detection (HPLC-UV-DAD). The differential expression of photosynthetic proteins (Rubisco and photosystem II) was analyzed by Western blotting. The contents of both chemical classes decreased during the reproductive stage, though the prevalence of diterpenes in *var. sylvestris* and flavonoids in *var. lingua* remained unchanged; furthermore, even when the plants are grown under the same geographic and environmental conditions, Rubisco expression in *var. lingua* is twice that of *var. sylvestris*. In var. *lingua*, photosystem II proteins are 10 % less expressed. The study reveals the circadian and seasonal fluctuations and, thus, prevalence of the two main compound classes in the examined varieties. The expression of the investigated photosynthetic proteins provides insights into the two varieties, supporting the prevalence of *var. lingua* in Cerrado areas and *var. sylvestris* in Atlantic Forest areas.

## Highlights

• Diterpenes in var. *sylvestris* and flavonoids in *var. lingua* prevail all year round.
• The contents of both chemical classes were reduced during the reproductive stage.
• Rubisco expression in v*ar. lingua* is twice that of *var. sylvestris*.
• The two *C. sylvestris* varieties can be differentiated by their chemical profiles.

## Graphical abstract

**Figure:**
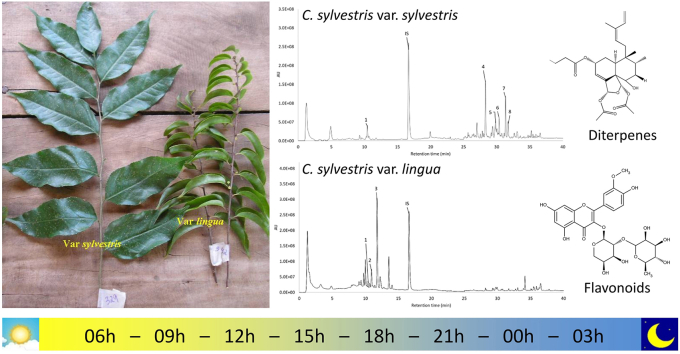

## 1 Introduction

*C. sylvestris* Sw. (Salicaceae) is a notable representative of the pantropical *Casearia* Jacq. genus, which contains approximately 180 species [1]. Due to its large chemical and morphological variations, this species constitutes a good example of the intersection between a high diversity of plant secondary metabolites and phenotypic plasticity [2,3]. One of the main characteristics of *C. sylvestris* is its high adaptive capacity, accounting for its large distribution in Africa and Central and South America, extending from Mexico to Argentina and Uruguay [1,2]. In Brazil, this species occurs in all biomes, covering a wide range of ecosystems and ecotones of the tropical Atlantic Forest, Amazon Forest, Cerrado (Savannah), Caatinga, and Pantanal [1,2,4].

Morphologically, *C. sylvestris* is characterized by large variations related to the tree size, leaf shape and texture, and number of inflorescences [5]. In general, the flowering stage diverges from July to November [6] and from June to August [7]. Because of its large morphological diversity, this species is classified into two distinct varieties [2]: *C. sylvestris* var. *sylvestris*, trees higher than 2 m with large dark green leaves; and *C. sylvestris* var. *lingua*, shrubs comprising light green coriaceous leaves [2,6].

The genetic diversity and structure of *C. sylvestris* populations were accessed using microsatellite markers comparing these factors among nine such populations from the Atlantic Forest and Cerrado areas and their ecotones. The morpho-anatomical and genetic data available on *C. sylvestris* indicate a close relationship between *var. lingua* and the Cerrado area, and *var. sylvestris* and the Atlantic Forest. These two varieties coexist in the Cerrado/Atlantic Forest ecotones, with individuals presenting intermediary characteristics [2,8]. They also show distinct chemical profiles; while *C. sylvestris* var. *sylvestris* produces mostly clerodane-type diterpenes, *C. sylvestris* var. *lingua* predominantly contains phenolic compounds [5,9,10].

In general, *C. sylvestris* and other species of the genus *Casearia* are characterized by the presence of highly oxygenated clerodane diterpenes, marked by unsaturations and esterified hydroxyl groups. These compounds, also called casearins, constitute a taxonomic marker for the genus *Casearia* and are widely found in the leaves, stems, stem barks, roots, and seeds of *Casearia* species [[11], [12], [13], [14], [15], [16]]. At least 153 clerodane-type diterpenes have been isolated from *Casearia* species, with 42 isolated from *C. sylvestris* [15]. The basic structure of such highly oxygenated and esterified compounds involves a decalin system (A and B rings) in a *cis* configuration bonded to a tetrahydrofuran ring (C; Fig. S1, Supporting information) [17].

In addition to their role as taxonomic markers in *C. sylvestris*, casearins are known for their pharmacological properties, as shown by related extracts, fractions, and isolated compounds. Thus far, phytochemical and pharmacological analyses have attributed anti-ulcer, anti-inflammatory, antiparasitic, cytotoxic, antimicrobial, and antitumor activities to the occurrence of clerodane-type diterpenes [15,16,[18], [19], [20], [21], [22], [23], [24]].

Along with the clerodane-type diterpenoids, *Casearia* species are characterized by the presence of phenolic compounds such as glycosylated flavonoids [16], ellagic acid and its derivatives [25], and gallic acid derivatives [26]. Fourteen 3-*O*-glycosylated flavonoids and one catechin have been identified in the leaves of *C. sylvestris* var. *lingua* [10].

Although the phytochemistry of *C. sylvestris* has been largely studied, one of the research gaps involves its metabolic dynamics, especially considering that the two varieties produce fundamentally different compound classes. Elucidating these dynamics is especially relevant since the therapeutic potential of *C. sylvestris* is influenced by its chemical profile. Considering that *C. sylvestris* has been traditionally used as a phytomedicine, one should note that the chemical profiles of the two *C. sylvestris* varieties investigated herein determine their corresponding biological activities. As recently evidenced by multivariate data analysis, *C. sylvestris* var. *lingua* collected in Cerrado areas possesses a chemical profile rich in glycosylated flavonoids and exhibits low cytotoxic activity and high antioxidant potential. *C. sylvestris* var. *sylvestris* from Atlantic Forest areas shows a chemical composition rich in clerodane-type diterpenoids and displays high cytotoxic activity and a low antioxidant potential [3].

Despite existing information on the geographical distribution, morphological traits, and chemical profile of *C. sylvestris*, the seasonal variation and circadian rhythms of casearins and phenolic compounds produced by the two *C. sylvestris* varieties have, to date, not been investigated. Notably, identifying circadian rhythms and seasonal trends is a challenging task. As reviewed for European trees, for example, the seasonal variation of different compound classes depends on the plant specificity, ontogeny, region, habitat, altitude, and climate pattern [27]. Generally, more attention has been given to studying fluctuations in the levels of phenolic compounds in tree leaves and essential oils from aromatic plants [[28], [29], [30]], while bioactive compounds from other secondary metabolite classes are mostly understudied [28]. Even regarding leaf phenolics, which are widespread across all plant species, seasonal fluctuations have remained largely uninvestigated [30].

The novelty of the current study, therefore, involves describing the metabolic dynamics of the two main metabolite classes biosynthesized by *C. sylvestris* var. *sylvestris* and *C. sylvestris* var. *lingua.* The objective was to investigate the seasonal and circadian variations in the contents of diterpenes and glycosylated flavonoids produced by these two varieties, both collected in the same geographical area. In this regard, their leaves were sampled monthly over 1 year, processed, extracted, and analyzed by high-performance liquid chromatography coupled to diode array detection (HPLC-UV-DAD). Considering previous studies on the prevalence of *var. lingua* in open and xeric environments and *var. sylvestris* in dense and humid forests, we also investigated the differential expression of photosynthetic proteins (Rubisco and photosystem II) in the samples collected for the present study.

## 2 Results and discussion

### 2.1 Morphological and chemical characterization of the two *C. sylvestris* varieties

**Fig. 1:**
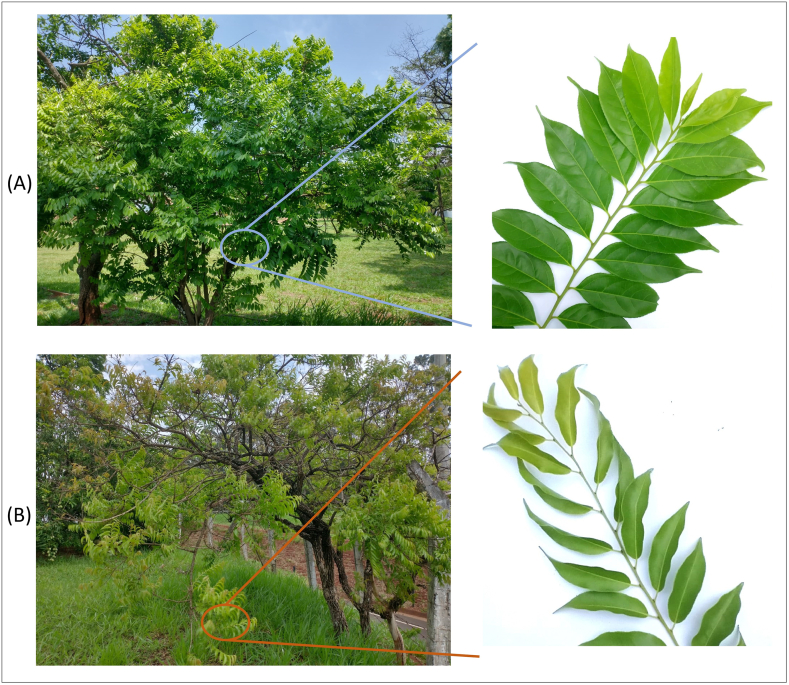
(A) *C. sylvestris* var. *sylvestris* and (B) *C. sylvestris* var. *lingua*, with representative branches showing the leaf morphology. Both individuals naturally occur in the same Brazilian Cerrado area and were sampled in the circadian and seasonal variation study. The photographs were taken on the same day.

**Fig. 2:**
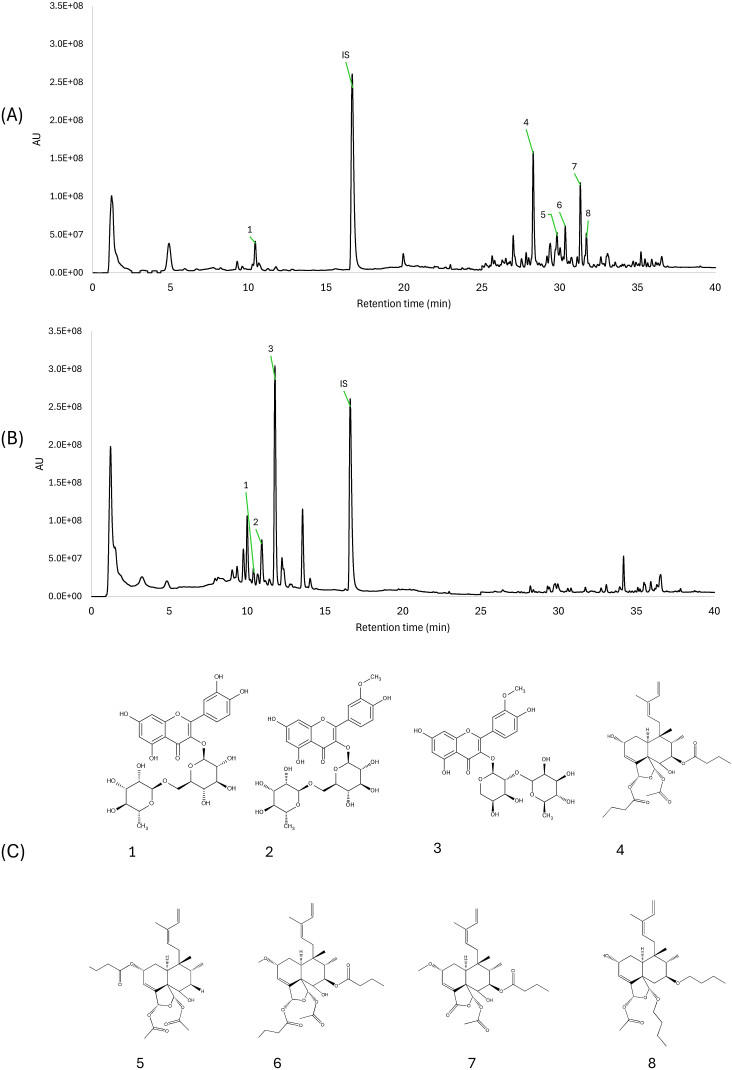
UHPLC-UV-DAD chromatograms of (A) *C. sylvestris* var. *sylvestris* and (B) *C. sylvestris* var. *lingua*, showing the compounds quantified in the present study. The chromatograms are plotted at 254 nm (from 0 to 25 min) and 235 nm (from 25 to 45 min), highlighting the elution regions of glycosylated flavonoids and clerodane-type diterpenes, respectively. Chromatographic conditions – chromatographic column: Kinetex 150 × 2.1 mm, 2.6 μm; mobile phase: water and acetonitrile (ACN), both containing 0.1 % of formic acid; gradient elution: 10–25 % ACN from 0 to 15 min, 25–90 % ACN until 35 min, and 90 % ACN until 40 min; flow rate: 400 μL min^−1^; oven temperature: 35 °C; and injection volume: 2 μL. (C) Chemical structures of the glycosylated flavonoids and clerodane-type diterpenes monitored in *C. sylvestris* var. *sylvestris* and *C. sylvestris* var. *lingua* over 12 months for the circadian and seasonal variation study. 1 = rutin, 2 = narcisin, 3 = isorhamnetin- 3-*O*-α-L-rhamnopyranosyl-(1 → 2)-α-L-arabinopyranoside (abbreviated as isorhamnetin-3-*O*-Gluc), 4 = casearin D, 5 = caseargrewiin F, 6 = casearin S, 7 = casearin I, 8 = casearin J, and IS = internal standard.

The morphological characterization of the two *C. sylvestris* varieties was critical in correctly assigning the individuals for the seasonal and circadian study. According to the literature, the main morphological characteristics differentiating the two *C. sylvestris* varieties are the sizes and overall aspects of the trees, and the shape and color of the leaves [6]. Fig. 1A and B shows the characteristic morphologies of the *C. sylvestris* var. *sylvestris* and *C. sylvestris* var. *lingua* trees and leaves monitored in the present study. Furthermore, the chemical profiles of the leaves of these two individuals also support the differentiation between the two varieties, as previously described [9,10]. The chromatogram of the hydro-alcoholic extract of the *var. sylvestris* leaves (Fig. 2A) confirms the existence of a region rich in diterpenes (with peaks eluting after the internal standard and a maximum ultraviolet [UV] spectral wavelength of 235 nm, characteristic of clerodane-type diterpenes). Likewise, the chromatogram in Fig. 2B confirms the characteristic *C. sylvestris* var. *lingua* chemical profile, rich in phenolic compounds (with peaks eluting before the internal standard and maximum UV spectral wavelengths of 256 and 355 nm) [9,10,17].

### 2.2 Circadian rhythms and seasonal variations

Aside from genetic influences, many of the qualitative and quantitative variations in the production of secondary metabolites by plants are associated with adaptations to the environment and the developmental stage. Phenotypic chemical variations can be related to ontogeny, phenological stages, circadian rhythms, seasonal cycles, and biotic and abiotic stresses [31].

To minimize environmental effects, the present study employed two *C. sylvestris* varieties grown in the same Cerrado area of São Paulo State, separated by a distance of 200 m. For documentation and monitoring purposes, temperature and relative humidity data were also compiled during the collection period in each month (Fig. S2, Supporting information). Samples were collected monthly, every 3 h within a 48-h period, over 1 year, after which they were processed, extracted, and analyzed. Based on comparisons with previously isolated authentic standards and UV, high-resolution mass spectrometry (HRMS), and tandem mass spectrometry (MS/MS) data, eight target compounds – including three glycosylated flavonoids and five diterpenes (Fig. 2C) – were assigned on the chromatograms (Fig. 2A and B) and quantified by HPLC-UV-DAD. Table 1 presents annotations of glycosylated flavonoids and diterpenes based on their HRMS, MS/MS, and UV data.

**Table 1:** Identification of glycosylated flavonoids and diterpenes in *C. sylvestris* var. *lingua* and *C. sylvestris* var. *sylvestris* based on their HRMS, MS/MS and UV data.

Peak No.	Compound name	Formula	*RR*t (min)	UV max (nm)	[M+Na]^+^ (*m*/*z*)	[M − H]^-^ (*m*/*z*)	MS/MS	Exact mass (calculated)	Error (ppm)
1	Quercetin-3-*O-*rutinoside (rutin)	C_27_H_30_O_16_	10.5	256 (265sh); 353	–	609.1486^a^	609 → 300 (301)	609.1461	−4.104
2	Isorhamnetin-3-O-rutinoside (narcisin)	C_28_H_32_O_16_	10.9	255 (267sh); 354	–	623.1665^a^	623 → 314 (315)	623.1618	−7.542
3	Isorhamnetin-3-*O*-α-L-rhamnopyranosyl-(1 → 2)-α-L-arabinopyranoside	C_27_H_30_O_15_	11.8	254 (265sh); 353	–	593.1553^a^	593 → 314 (315)	593.1512	−6.912
4	Casearin D	C_30_H_44_O_9_	28.3	236	571.2849^b^	–	571 → 483, 423	571.2878	5.076
5	Caseargrewiin F	C_28_H_40_O_8_	29.8	230	527.2615^b^	–	527 → 467, 407	527.2615	0.000
6	Casearin S	C_27_H_38_O_7_	30.3	225	497.2517^b^	–	497 → 437, 409	497.2510	−1.408
7	Casearin I	C_30_H_44_O_8_	31.3	225	555.2934^c^	–	–	555.2928	−1.081
8	Casearin J	C_31_H_46_O_9_	31.8	235	585.3027^b^	–	585 → 497, 437	585.3034	1.196

a Assigned based on micrOTOF QII (ESI-TOF) data.

b Assigned based on micrOTOF Q II (ESI-TOF) and LC-DAD-ESI-Orbitrap data.

c Assigned based on LC-DAD-ESI-Orbitrap data. All assignments were done using previously isolated standards.

The results indicate that the concentration of clerodane diterpenes in *var. sylvestris* is consistently higher (throughout each day and on all days of the year) than that of the flavonoids, as we hypothesized, given previous information on the chemospecificity of the two varieties [5,9,10]. On the other hand, flavonoids prevail over diterpenes in *var. lingua*.

**Fig. 3:**
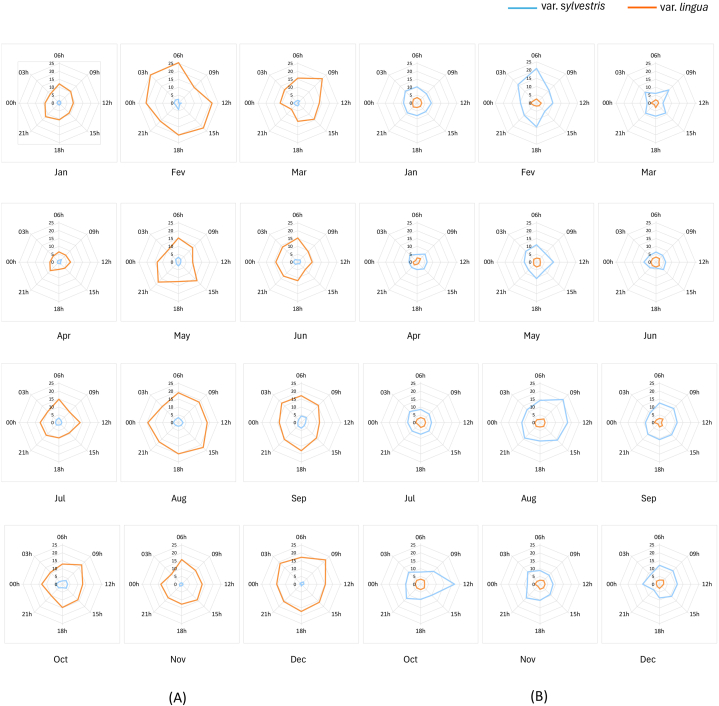
Comparison of the monthly circadian variations of the sum of (A) glycosylated flavonoids and (B) clerodane-type diterpenes in *C. sylvestris* var. *sylvestris* and *C. sylvestris* var. *lingua* over 1 year*.* The content of each compound was quantified in mg·g^−1^ dry weight using a previously validated chromatographic method (Bueno et al., 2015) [9].

Statistical analysis of the circadian oscillations was conducted considering the measurements of the samples collected in the daytime and nighttime. Daytime sampling was performed at 9:00, 12:00, 15:00, and 18:00, totaling eight measurements over 2 consecutive days; nighttime sampling was conducted at 21:00, 00:00, 3:00, and 6:00, also constituting eight measurements over 2 consecutive days. These two groups were compared using the Mann–Whitney test (sample size = 8, α = 0.05), with the comparisons performed for all 12 months, separately, for the total glycosylated flavonoids and clerodane diterpenes quantified in the two varieties. Although oscillations were observed in the circadian rhythms for the three flavonoids and five diterpenes, only a few statistically significant alterations were identified for some compounds considering the day/night cycles (Table S1, Supporting information). Fig. 3A and B depict the monthly oscillation patterns of each class of compounds over 1 year for both varieties, and Fig. S3 (Supporting information) shows the circadian rhythms of the glycosylated flavonoids and clerodane-type diterpenes in both varieties separately.

Notably, the production rhythm of secondary metabolites and the physiological manifestations of biological clocks are still far from fully elucidated, despite the increasingly precise genetic and biochemical characterization of oscillating units in organisms [32]. In plants, circadian rhythms control essential functions such as gene expression, stomatal opening, and the timing component of photoperiodism [33]. They also directly regulate metabolism, which is related to plant development, growth, reproduction, and metabolite products [34]. However, the contents of both secondary and primary metabolites in plants are not stable and can differ significantly between species and from the same biosynthetic pathways [27].

Considering that the circadian production of flavonoids and diterpenes biosynthesized by *C. sylvestris* did not differ significantly in the day/night cycles, the findings confirm the prevalence of the production of glycosylated flavonoids by *var. lingua* and diterpenes by *var. sylvestris*, even with the observed oscillations.

**Fig. 4:**
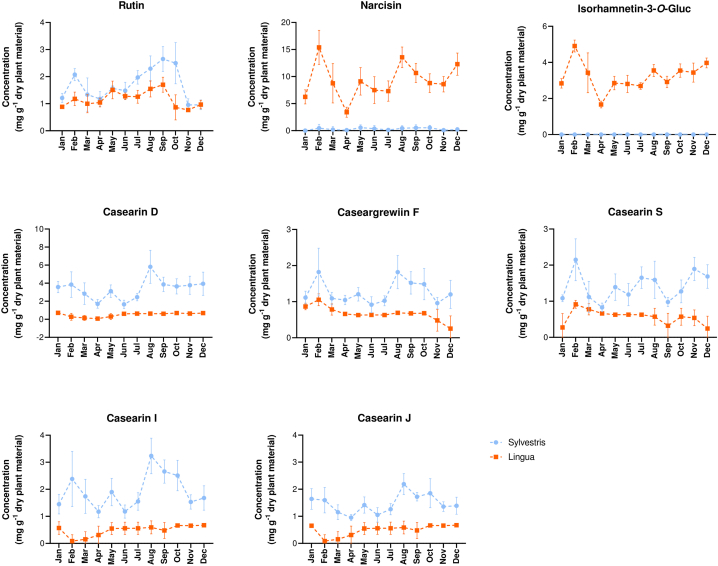
Seasonal variation of glycosylated flavonoids and clerodane-type diterpenes in *C. sylvestris* var. *sylvestris* and *C. sylvestris* var. *lingua.* Each month corresponds to the average contents of each compound quantified in the leaves (mg·g^−1^ dry weight) from 16 sampling measurements (n = 16) taken over 48 h. The error bar represents the standard deviation (SD) of the 16 replicates.

Aiming to contribute to elucidating the seasonal variations of tropical plant species, we focused on the biosynthesis of flavonoids by *var. lingua* and clerodane diterpenes by *var. sylvestris*, separately, in the seasonal variation study of *C. sylvestris*. Regarding the glycosylated flavonoids in *var. lingua*, the same seasonal oscillation pattern was observed for the three flavonoids under investigation. The contents of rutin, narcisin, and isorhamnetin-3-*O*-α-L-rhamnopyranosyl-(1 → 2)-α-L-arabinopyranoside tend to decrease, especially in April (Fig. 4). In var. *sylvestris*, the same pattern was observed for rutin, the only flavonoid that could be detected and quantified in this variety. Interestingly, comparing the rutin content in both varieties, no statistically significant differences were observed (Table S2).

Trace amounts of the clerodane diterpenes were detected in *var. lingua*. However, for the five compounds monitored in *var. sylvestris*, one can note the same oscillation pattern observed in the flavonoids. There is a decrease in the content of the five casearins, especially in April, and in mid-August, the relative contents are restored. This is notable, given that the field observations show that the flowering period begins in May, with production peaks in June. Until mid-August, it was still possible to observe flowers coexisting with fruits. This suggests that the lowest levels of all studied metabolites coincide with the plant's reproductive phase.

**Fig. 5:**
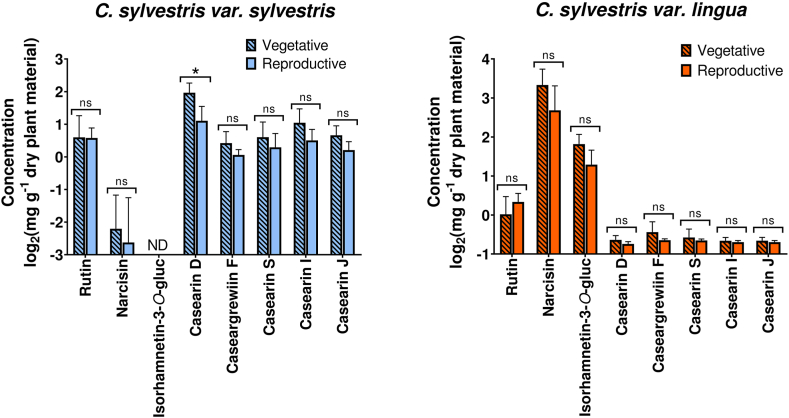
Results of the Mann–Whitney test, comparing the contents of all investigated compounds measured at the collection time points during the vegetative (August to March, n = 128) and reproductive (April to July, n = 64) stages. (∗) Significant difference (p-value <0.05). (ns) Non-significant difference (p-value <0.05). (A) *C. sylvestris* var. *sylvestris*. (B) *C. sylvestris* var. *lingua*. *ND = not detected*.

The results are supported by insights into the phenological events in plants, which is a dominant aspect of plant ecology. The timing of the change between the vegetative and reproductive phases that occurs in the flowering period is crucial for seed development, both for individuals and populations [35]. In tropical ecosystems, phenology may be less sensitive to temperature and photoperiods and influenced more by seasonal changes in rainfall [35]. In the location where the present study was conducted, the rainfall period is more prominent from October to March, which also coincides with the highest temperatures (Fig. S2). This corresponds to the observed *C. sylvestris* vegetative stage, during which the average flavonoid and diterpene contents are higher in both varieties, except for rutin in *var. lingua*. Although this tendency can be observed for all compounds, the results of the Mann–Whitney test, comparing the contents of all investigated compounds between the vegetative (August to March) and reproductive (April to July) stages, are significant only for casearin D in *var. sylvestris* (p < 0.05; Fig. 5).

### 2.3 Expression of photosynthetic proteins in the two *C. sylvestris* varieties

Available data on the distribution of *C. sylvestris* in many different biomes suggest that *var. lingua* is better adapted to xeric and open environments and, therefore, more exposed to sunlight. In contrast, *var. sylvestris* is mainly found in areas of low light intensity, with significant canopy influence [2,3]. Corresponding to this observation, the light green color of *var. lingua* leaves and the dark green color of *var. sylvestris* leaves suggest a possible adaptation of the photosynthetic apparatus to the high levels of sunlight to which these plants are exposed. In general, opposed to sun leaves, shade leaves have been reported to maximize light capture. These leaves are thin and light and show high chlorophyll concentrations per unit leaf mass and low ATPase activities and Rubisco contents compared to their sun leaf counterparts [36]. Furthermore, depending on the light intensity to which plants are exposed, different molecular adaptation strategies occur synchronously to modulate the rate of light absorption [37]. In plants exposed to low light intensities, photosynthesis tends to be limited by the capacity of the system to generate the ribulose-1,5-bisphosphate (RuBP) substrate for Rubisco, which is dependent on the production of ATP and NADPH molecules provided by thylakoid reactions in the light [38].

**Fig. 6:**
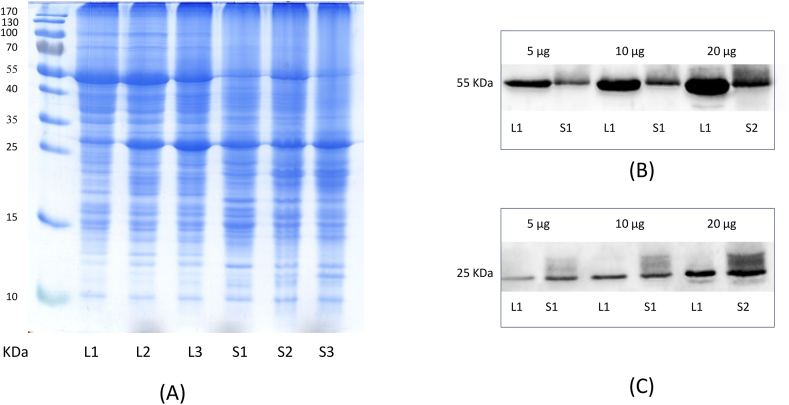
(A) SDS-PAGE analysis of proteins extracted from the two *C. sylvestris* varieties (uncropped and unprocessed). Lanes L1, L2, and L3 are replicates of protein extracts from *var. lingua*, and lanes S1, S2, and S3 are replicates of protein extracts from *var. sylvestris.* (B) Western blotting analysis of the abundance of the Rubisco large subunit (55 kDa). (C) Western blotting analysis of the abundance of the P25K (PSII) protein (25 kDa). In (B) and (C), the samples were applied at three levels (5, 10, and 20 μg). L1 = representative sample of *var. lingua*, and S1 = representative sample of *var. sylvestris*.

Considering that the individuals from both varieties used in the present study grow naturally in the same location in a Cerrado area of São Paulo State, we included an analysis of the photosynthetic proteins, aiming to provide data to support the differentiation of the two varieties at the protein level. The leaves of five individuals of each variety were pooled together, and the proteins were quantified using the Bicinchoninic acid (BCA) assay. We also extracted and separately analyzed the two individuals monitored during the circadian and seasonal study. In total, three protein extracts of *var. sylvestris* and *var. lingua* were evaluated. The protein content ranges from 4.25 to 7.27 μg μL^−1^ considering all replicates from both varieties and was used for normalization prior to the sodium dodecyl sulfate-polyacrylamide gel electrophoresis (SDS-PAGE) separation (Fig. 6A).

From the resultant SDS-PAGE gel (Fig. 6A), one can observe a distinct protein pattern in the two varieties. The gel shows the characteristic region of Rubisco in the range of 55 kDa, noticeably higher in the protein extracts of *var. lingua* compared to the three *var. sylvestris* replicates. The expression of proteins in the range of 25–35 kDa, corresponding to the light-harvesting complex proteins (LHCII, corresponding to photosystem II), is rather similar in both varieties. The expression of these two main groups of proteins was analyzed by Western blotting, confirming greater levels of Rubisco in var. *lingua* compared to *var. sylvestris* (Fig. 6B). In contrast, *var. sylvestris* shows slightly greater levels of photosystem II proteins (Fig. 6C). Further relative quantifications by densitometry (n = 3, three concentration levels) highlight a twofold greater expression of Rubisco and a 10 % lower expression of photosystem II proteins in *var. lingua* compared to *var. sylvestris* (Fig. S4, Supporting information). This finding is also supported by the literature, as the antenna complex (comprising the LHC proteins, especially those assembled in photosystem II) of sun plants is relatively minor in comparison to that of plants that grow under shaded conditions. It is known that this complex is reduced by the antenna proteolytic enzymes (also ATP-dependent) that degrade the outer portion of the LHCII complex [37]. Interestingly, this corroborates the observations of the prevalence of flavonoids in *var. lingua*. Studies have shown that sun leaves synthesize higher amounts of phenolic compounds than leaves grown under shaded conditions [30]. Under these circumstances, flavonoids, besides acting as catalysts in the light phase of photosynthesis, play an important role in UV protection due to their UV-B absorbing capacity and antioxidant properties, which counteract harmful reactive oxygen species (ROS) formed during light stress [39].

## 3 Conclusions

The present study provides insights into the dynamics of the two main compound classes biosynthesized by the tropical plant species *C. sylvestris* by investigating the fluctuations of five diterpenes and three glycosylated flavonoids in two varieties. Considering previous studies describing the widespread presence of glycosylated flavonoids in *C. sylvestris* var. *lingua* and diterpenes in *C. sylvestris* var. *sylvestris*, the present study provides combined evidence on the distinct and constant chemical profile over the circadian cycle, with the seasonal variations showing a clear pattern of the reduced content of both chemical classes during the reproductive phase. The results on the expression of the two photosynthetic proteins represent another aspect for the differentiation between the two varieties, supporting the prevalence of *var. lingua* in Cerrado areas and *var. sylvestris* in Atlantic Forest areas. Although limited to 12 months of study and sampling the leaves of one individual of each variety every 3 h over a 48-h period per month, the present study provides new data regarding the metabolic dynamics of the two main compound classes biosynthesized by *C. sylvestris*. Further studies including a larger number of individuals, different locations, and 2 or more consecutive years will contribute to validating the findings of this study.

## 4 Experimental

### 4.1 Chemicals and reagents

Acetonitrile, ethanol, and isopropanol (J. T. Baker, USA) of chromatographic grade were used for the chemical analysis and sample extraction. Ultrapure water was obtained using a Millipore Milli-Q water purification system (Millipore, USA). Butyl gallate was purchased from Sigma-Aldrich (Germany). Narcisin, rutin, casearin D, casearin I, casearin S, and caseargrewiin F were previously isolated, purified, and identified in our laboratory [10]. Acetone, methanol, phenol, ammonium acetate, sodium dodecyl sulfate, sucrose, 2-mercaptoethanol, trichloroacetic acid, and Tris-HCl buffer (0.1 M, pH 8.0) of analytical grade were purchased from Sigma-Aldrich or Thermo Fisher Scientific (Germany) for protein extraction. The Pierce BCA Protein Assay Kit from Thermo Scientific (Germany) was used for protein quantification. A PageRuler prestained protein ladder 10–170 kDa (Pierce Protein Biology Products, Thermo Scientific) was used for SDS-PAGE and Western blotting, a nitrocellulose membrane (Hybond ECL 0.45 μm, 300 mm × 3 m, Amersham GE Healthcare, Sweden) was employed for ECL Western blotting, and an Ultrafin liquid B/W negative developer and fixer (Tetenal Superfix Plus Rapid Fixer, Germany) was used for autoradiography. The antibodies anti-RbcL (Rubisco large subunit), anti-P25K (LHCII – light-harvesting protein, photosystem II), and anti-rabbit IgG were acquired from Agrisera AB (Sweden).

### 4.2 Plant material

Prior to the circadian and seasonal variation investigation, five trees of each *C. sylvestris* variety were sampled in different locations of Araraquara, São Paulo State, Brazil (coordinates: 21.8190791 S, −48.1992606 W) in November 2012 between 7 and 9 a.m. The identities and assignments of the varieties were confirmed by Prof. Dr. Roseli Buzanelli Torres from the Instituto Agronômico de Campinas (IAC) in São Paulo State. Voucher specimens were deposited at the Herbarium of the IAC (IAC 55839 - ARA 13, P.C.P. Bueno 56 for *C. sylvestris* var. *lingua* and IAC 55840 - ARA 14, P.C.P. Bueno 57 for *C. sylvestris* var. *sylvestris*). After confirming the chemical profile of each sampled individual (Fig. S5, Supporting information), one tree of each variety was selected and marked for the circadian and seasonal variation study, taking into consideration the need for permanent, safe, and easy access to the trees over 1 year and an absence of artificial light, especially at night. The phenological events and the temperature and relative humidity were recorded during this process (Fig. S2). The protocol involved collecting the leaves of one branch (2 m from the ground) of each tree every 3 h within a 48-h period per month over 12 months. The branches, still attached to the trees, were washed with distilled water and dried with tissue paper. The leaves were then collected in a 50-mL Falcon tube, immediately frozen in liquid nitrogen, and kept at −80 °C until freeze-dried. The freeze-dried leaves were then ground using an analytical mill (A11 Basic, Ika LabortechniK, Germany) and aliquoted for subsequent extraction and chromatographic analysis [9]. All five trees of *C. sylvestris* var. *sylvestris* and *C. sylvestris* var. *lingua* were used in the photosynthetic protein analysis. The leaves of four branches of each of the five trees were collected, washed with distilled water, dried with tissue paper, immediately frozen in liquid nitrogen, and kept at −80 °C until protein extraction.

### 4.3 Secondary metabolite analysis

#### 4.3.1 Sample preparation

The dried leaves of each sample taken during the circadian and seasonal variation study were individually analyzed. For the sample extraction and preparation, 50 mg of each powdered sample was extracted with 1 mL of an extraction solvent – water/ethanol/isopropanol (50:30:20 %v/v) containing butyl gallate (0.5 mg mL^−1^) as an internal standard [9]. The samples were sonicated at room temperature for 30 min and then centrifuged at 5000×*g* for 5 min. Finally, 0.7 mL of the supernatants were taken and filtered through a 0.22-μm PTFE Millipore filter directly into 1.5-mL vials and immediately subjected to chromatographic analysis.

#### 4.3.2 Instrumentation and chromatographic parameters

Aliquots (2 μL) of each extracted sample were analyzed by UHPLC-UV-DAD (Ultimate 3000, Dionex) using a Kinetex C18 column (150 × 2.1 mm, 2.6 μm, 100 Å, Phenomenex), protected by a guard column from the same stationary phase. The chromatographic method has been previously developed and validated by Bueno et al. (2015) [9]. Briefly, the mobile phase consisted of purified water and acetonitrile (ACN) containing 0.1 % of formic acid. The separations were achieved under the following gradient elution: 10–25 % ACN from 0 to 15 min, 25–90 % ACN until 35 min, 90 % ACN until 40 min, returning to the initial condition of 10 % ACN within 2 min, and holding this concentration for a further 3 min. The flow rate and oven temperature were set to 400 μL min^−1^ and 35 °C, respectively, and the spectral absorption data were collected within 45 min over the 200–800 nm wavelength range. Chromatograms were plotted at 235 and 254 nm, and the main peaks were identified by comparison with the retention times and UV spectra of previously isolated standards. Likewise, a representative sample was subjected to HRMS and MS/MS (micrOTOF-Q II, Bruker Daltonics and Exactive Plus Orbitrap, Thermo Scientific) to confirm the peak assignments, using the HRMS parameters previously described by Bueno et al. (2016) [10] and Danuello et al. (2020) [17].

#### 4.3.3 Quantitative analysis of glycosylated flavonoids and clerodane-type diterpenes

Three glycosylated flavonoids – rutin, narcisin, and isorhamnetin- 3-*O*-α-L-rhamnopyranosyl-(1 → 2)-α-L-arabinopyranoside (abbreviated as isorhamnetin-3-*O*-Gluc) – and five clerodane-type diterpenes (casearin D, caseargrewiin F, casearin S, casearin I, and casearin J) were quantified individually in the circadian and seasonal variation study. Based on the characteristic chromophores and assuming similar molar extinction coefficients for each compound class, the relative quantities of the target secondary metabolites were analyzed using the analytical curves (n = 3) of narcisin (254 nm), representing the glycosylated flavonoids, and caseargrewiin F (235 nm), representing the clerodane-type diterpenes. Standard solutions of narcisin and caseargrewiin F were prepared using a 50:30:20 (%v/v) mixture of water/ethanol/isopropanol, containing the internal standard (butyl gallate, 0.5 mg mL^−1^), followed by serial dilution to achieve concentrations ranging from 10.0 to 800.0 mg mL^−1^ for narcisin and 5.0–400 mg mL^−1^ for caseargrewiin F. The obtained linear correlation coefficients are 0.999 for narcisin (y = 0.002 x + 0.014) and 0.999 for caseargrewiin F (y = 0.007 x + 0.041). The results are expressed in mg·g^−1^ dry weight.

#### 4.3.4 Statistical and data analysis

The chromatographic data involving the peak areas of the eight targeted compounds were exported to Microsoft Excel 2010 (Microsoft, USA); the chromatographic signals were exported at 254 nm (from 0 to 25 min) and 235 nm (from 25 to 45 min) and normalized against the internal standard (254 nm). The content of each compound was calculated in mg·g^−1^ using the calibration curves of rutin and caseargrewiin F. Statistical analyses were performed using Prism software (version 8.0.2; GraphPad software©, La Jolla, CA, USA). A student's t-test was used to compare the means for data following a normal distribution, and the Mann–Whitney test was employed for non-normally distributed data. The Anderson–Darling, D'Agostino–Pearson omnibus, and Shapiro–Wilk methods were employed to assess the normality of the data distribution. Multiple *t*-test analyses were performed with fewer assumptions, not assuming consistent standard deviations (SDs), and their p-values were corrected using the Holm–Sidak method. The correlations were assessed using Spearman correlation analysis due to the non-normality of the data distributions.

### 4.4 Photosynthetic protein analysis

#### 4.4.1 Protein extraction

Three representative samples of each *C. sylvestris* variety were utilized for protein extraction. Samples L1 and S1 comprised only one tree of each variety, and the same trees were selected for the circadian and seasonal variation study. Samples L2 and L3 (*var. lingua*) and S2 and S3 (*var. sylvestris*) included the leaves from the five sampled trees of each variety. Aliquots of 12 g of each representative sample (L1, L2, L3, S1, S2, and S3) were ground using an analytical mill and liquid nitrogen. Subsequently, 5 g of the obtained powders were extracted according to the procedure described by Wang et al. (2006) [40], using TCA/acetone and methanol washes followed by a phenol extraction; the only modification involved the sample amount. After drying, 5 mg of each protein extract was transferred to a 1.5-mL Eppendorf tube, and the proteins were resuspended in 0.5 mL of HEPES buffer solution (5 mM, pH 7.5, 10 mM EDTA) and stored at −80 °C. The protein concentrations were measured using the BCA assay kit (Pierce BCA Protein Assay Kit, Thermo Scientific; n = 3).

#### 4.4.2 SDS-PAGE and Western blotting

For the qualitative analyses, 10 μg of the proteins from all samples were loaded on an SDS-PAGE gel, separated according to the method described by Laemmli (1970) [41], and stained with Coomassie Blue. The concentration of the SDS-PAGE gel was 4 % for the stacking gel (running at 10 mA for 60 min) and 13 % for the separation gel (running at 20 mA for 90 min). For the Western blotting analyses, 5, 10, and 20 μg of protein from samples L1 and S1 were separated by SDS-PAGE and then transferred to nitrocellulose membranes (Hybond ECL 0.45 μm, Amersham GE Healthcare) overnight at 80 mA. The membranes were blocked in 2 % skimmed milk in phosphate-buffered saline (PBS) buffer at pH 7.2–7.4 (3 × 15 min) and probed with the polyclonal antibody anti-RbcL (1:5000) and anti-P25K (1:3000). After an incubation time of 96 h, the membranes were washed three times with PBS buffer and probed with a second anti-rabbit IgG antibody (1:10000) over 2 h. The antibody was then detected by enhanced chemiluminescence, and the relative amounts of each protein subunit were estimated by densitometry (Fusion SL, Peqlab Biotechnologie, Germany).

## CRediT authorship contribution statement

**Paula C.P. Bueno:** Writing – review & editing, Writing – original draft, Project administration, Methodology, Investigation, Formal analysis, Data curation. **Gabriel S. Viana:** Software, Methodology, Investigation. **Livia L. Thomaz:** Methodology, Investigation. **Daniela A. Chagas-Paula:** Writing – review & editing. **Michael Hippler:** Supervision, Project administration, Formal analysis, Conceptualization. **Alberto J. Cavalheiro:** Writing – review & editing, Supervision, Project administration, Formal analysis, Conceptualization.

## Declaration of competing interest

The authors declare that they have no known competing financial interests or personal relationships that could have appeared to influence the work reported in this paper.

## References

[1] Marquete R., Torres R.B. (2022). A new species of Casearia Jacq. from Brazil. Rodriguesia.

[2] Cavallari M.M., Gimenes M.A., Billot C., Torres R.B., Zucchi M.I., Cavalheiro A.J. (2010). Population genetic relationships between Casearia sylvestris (Salicaceae) varieties occurring sympatrically and allopatrically in different ecosystems in south-east Brazil. Ann. Bot..

[3] Bueno P.C.P., Abarca L.F.S., Anhesine N.B., Giffoni M.S., Pereira F.M.V., Torres R.B., Souza R.W.R., Ferreira P.M.P., Pessoa C., Cavalheiro A.J. (2021). Infraspecific chemical variability and biological activity of Casearia sylvestris from different Brazilian biomes. Planta Med..

[4] Carvalho P.E.R. (2007). https://ainfo.cnptia.embrapa.br/digital/bitstream/CNPF-2009-09/42433/1/Circular138.pdf.

[5] Claudino J.C., Sacramento L.V.S., Koch I., Santos H.A., Cavalheiro A.J., Tininis A.G., Santos A.G. (2013). Evaluation of morpho-anatomical and chemical differences between varieties of the medicinal plant Casearia sylvestris Swartz. Ann. Acad. Bras. Cienc..

[6] Klein R.M., Sleumer H.O., Reitz R. (1984). Herbário Barbosa Rodrigues, Itajaí.

[7] Lorenzi H. (1992).

[8] Cavallari M.M., Billot C., Bouvet J.M., Favreau B., Zucchi M.I., Palmieri D.A., Gimenes M.A. (2008). Isolation and characterization of microsatellite markers for Casearia sylvestris Sw. (Salicaceae), a neotropical medicinal tree. Mol. Ecol. Resour..

[9] Bueno P.C.P., Pereira F.M.V., Torres R.B., Cavalheiro A.J. (2015). Development of a comprehensive method for analysing clerodane-type diterpenes and phenolic compounds from Casearia sylvestris Swartz (Salicaceae) based on ultra high-performance liquid chromatography combined with chemometric tools. J. Sep. Sci..

[10] Bueno P.C.P., Passareli F., Anhesine N.B., Torres R.B., Cavalheiro A.J. (2016). Flavonoids from Casearia sylvestris swartz variety lingua (salicaceae). Biochem. Syst. Ecol..

[11] Carvalho P.R.F., Furlan M., Young M.C.M., Kingston D.G.I., Bolzani V.S. (1998). Acetylated DNA-damaging clerodane diterpenes from Casearia sylvestris. Phytochemistry.

[12] Santos A.G., Perez C.C., Tininis A.G., Bolzani V.S., Cavalheiro A.J. (2007). Clerodane diterpenes from leaves of Casearia sylvestris Swartz. Quim. Nova.

[13] Carvalho E.S., Santos A.G., Cavalheiro A.J. (2009). Identificação de diterpenos clerodânicos em diferentes órgãos de Casearia sylvestris Swartz. Rev. Ciênc. Farm. Básica Apl.

[14] Wang W., Ali Z., Li X.C., Smillie T.A., Guo D.A., Khan I.A. (2009). New clerodane diterpenoids from Casearia sylvestris. Fitoterapia.

[15] Santos A.G., Ferreira P.M.P., Vieira G.M., Perez C.C., Tininis A.G., Silva G.H., Bolzani V.S., Costa-Lotufo L.V., Pessoa C.D.Ó., Cavalheiro A.J. (2010). Casearin X, its degradation product and other clerodane diterpenes from leaves of Casearia sylvestris: evaluation of cytotoxicity against normal and tumor human cells. Chem. Biodivers..

[16] Xia L., Guo Q., Tu P., Chai X. (2015). The genus Casearia: a phytochemical and pharmacological overview. Phytochem. Rev..

[17] Danuello A., Castro R.C., Pilon A.C., Bueno P.C.P., Pivatto M., Vieira Júnior G.M., Carvalho F.A., Oda F.B., Perez C.J., Lopes N.P., Santos A.G., Ifa D.R., Cavalheiro A.J. (2020). Fragmentation study of clerodane diterpenes from Casearia species by tandem mass spectrometry (quadrupole time-of-flight and ion trap). Rapid Commun. Mass Spectrom..

[18] Ferreira P.M.P., Santos A.G., Tininis A.G., Costa P.M., Cavalheiro A.J., Bolzani V.S., Moraes M.O., Costa-Lotufo L.V., Montenegro R.C., Pessoa C. (2010). Casearin X exhibits cytotoxic effects in leukemia cells triggered by apoptosis. Chem. Biol. Interact..

[19] Ferreira P.M.P., Costa-Lotufo L.V., Moraes M.O., Barros F.W., Martins A.M., Cavalheiro A.J., Bolzani V.S., Santos A.G., Pessoa C. (2011). Folk uses and pharmacological properties of Casearia sylvestris: a medicinal review. Ann. Acad. Bras. Cienc..

[20] Ferreira P.M.P., Bezerra D.P., Silva J.N., Costa M.P., Ferreira J.R.O., Alencar N.M.N., Figueiredo I.S.T., Cavalheiro A.J., Machado C.M.L., Chammas R., Alves A.P.N.N., Moraes M.O., Pessoa C. (2016). Preclinical anticancer effectiveness of a fraction from Casearia sylvestris and its component casearin x: in vivo and ex vivo methods and microscopy examinations. J. Ethnopharmacol..

[21] Ribeiro S.M., Fratucelli E.D.O., Bueno P.C.P., Castro M.K.V., Francisco A.A., Cavalheiro A.J., Klein M.I. (2019). Antimicrobial and antibiofilm activities of Casearia sylvestris extracts from distinct Brazilian biomes against Streptococcus mutans and Candida albicans. BMC Complement. Altern. Med..

[22] Spósito L., Oda F.B., Vieira J.H., Carvalho F.A., Ramos M.A.R., Castro R.C., Crevelin E.J., Crotti A.E.M., Santos A.G., Silva P.B., Chorilli M., Bauab T.M. (2019). In vitro and in vivo anti-helicobacter pylori activity of Casearia sylvestris leaf derivatives. J. Ethnopharmacol..

[23] Santos A.L., Amaral M., Hasegawa F.R., Lago J.H.G., Tempone A.G., Sartorelli P. (2021). (-)-T-Cadinol - a sesquiterpene isolated from Casearia sylvestris (Salicaceae) – displayed in vitro activity and causes hyperpolarization of the membrane potential of Trypanosoma cruzi. Front. Pharmacol..

[24] Ribeiro S.M., Bueno P.C.P., Cavalheiro A.J., Klein M.I. (2023). Effect of extracts, fractions, and isolated molecules of Casearia sylvestris to control Streptococcus Mutans cariogenic Biofilm. Antibiotics.

[25] Silva S.L., Calgarotto A.K., Chaar J.S., Marangoni S. (2008). Isolation and characterization of ellagic acid derivatives isolated from Casearia sylvestris SW aqueous extract with anti-Pla2 activity. Toxicon.

[26] Silva S.L., Chaar J.S., Yano T. (2009). Chemotherapeutic potential of two gallic acid derivative compounds from leaves of Casearia sylvestris Sw (Flacourtiaceae). Eur. J. Pharmacol..

[27] Formato M., Scharenberg F., Pacifico S., Zidorn C. (2022). Seasonal variations in phenolic natural products in Fagus sylvatica (European beech) leaves. Phytochemistry.

[28] Zidorn C. (2018). Seasonal variation of natural products in European trees. Phytochem. Rev..

[29] Li Y., Zidorn C. (2022). Seasonal variations of natural products in European herbs. Phytochem. Rev..

[30] Elghani E.M.A., El Sayed A.M., Emam M.M.A., Al-Mahallawi A.M., Tadros S.H., Soliman F.M., Youssef F.S. (2023). Seasonal metabolic profiling of Valencia orange leaf essential oil using GC coupled with chemometrics, nano-formulation, and insecticidal evaluation: in vivo and in silico. RSC Adv..

[31] Moore B.D., Andrew R.L., Külheim C., Foley W.J. (2014). Explaining intraspecific diversity in plant secondary metabolites in an ecological context. New Phytol..

[32] Rascher U., Hü M.T., Siebke K., Osmond B., Beck F., Lü U. (2001). Spatiotemporal variation of metabolism in a plant circadian rhythm: the biological clock as an assembly of coupled individual oscillators. Proc. Natl. Acad. Sci..

[33] Dodd A.N., Salathia N., Hall A., Kévei E., Tóth R., Nagy F., Hibberd J.M., Millar A.J., Webb A.A.R. (2005). Molecular biology: genome-scale identification of nucleosome positions in S. cerevisiae. Science.

[34] Kim J.A., Kim H.S., Choi S.H., Jang J.Y., Jeong M.J., Lee S.I. (2017). The importance of the circadian clock in regulating plant metabolism. Int. J. Mol. Sci..

[35] Cleland E.E., Chuine I., Menzel A., Mooney H.A., Schwartz M.D. (2007). Shifting plant phenology in response to global change. Trends Ecol. Evol..

[36] Mathur S., Jajoo A.J.L. (2018). Photosynthetic efficiency in sun and shade plants. Photosynthetica.

[37] Ruban A.V. (2009). Plants in light. Commun. Integr. Biol..

[38] Gurrieri L., Sparla F., Zaffagnini M., Trost P. (2024). Dark complexes of the Calvin-Benson cycle in a physiological perspective. Semin. Cell Dev. Biol..

[39] Harborne J.B., Williams C.A. (2000). Advances in flavonoid research since 1992. Phytochemistry.

[40] Wang W., Vignani R., Scali M., Cresti M. (2006). A universal and rapid protocol for protein extraction from recalcitrant plant tissues for proteomic analysis. Electrophoresis.

[41] Laemmli U.K. (1970). Cleavage of structural proteins during the assembly of the head of bacteriophage T4. Nature.

