# Supplementary material for: Seasonal and circadian rhythms of clerodane diterpenes and glycosylated flavonoids in two varieties of Casearia sylvestris Sw. (Salicaceae)
Source: Heliyon. 2024 Oct 17;10(20):e39488. doi: 10.1016/j.heliyon.2024.e39488 (PMC11513561; doi:10.1016/j.heliyon.2024.e39488)
Supplement: Multimedia component 1 [file mmc1.pdf]

Supplementary information

Seasonal and circadian rhythms of clerodane diterpenes and glycosylated flavonoids in two varieties of *Casearia sylvestris* Sw. (Salicaceae)

Paula C. P. Bueno [a, b, c*], Gabriel S. Viana [b], Livia L. Thomaz [a], Daniela A. Chagas-Paula [b], Michael Hippler [d], Alberto J. Cavalheiro [a]

[a] Institute of Chemistry, São Paulo State University, UNESP, Francisco Degni 55, 14800-900, Araraquara/SP, Brazil

[b] Institute of Chemistry, Federal University of Alfenas, UNIFAL, Gabriel Monteiro da Silva 700, 37130-001, Alfenas/MG, Brazil

[c] Leibniz Institute of Vegetable and Ornamental Crops, IGZ, Theodor-Echtermeyer-Weg 1, 14979, Großbeeren, Germany

[d] Institut für Biologie und Biotechnologie der Pflanzen (IBBP), Westfälische Wilhelms-Universität Münster, Schlossplatz 8, 48143, Münster, Germany

*Corresponding author:

Prof. Dr. Paula Carolina Pires Bueno

bueno@igzev.de

Present address: Leibniz Institute of Vegetable and Ornamental Crops, IGZ, Theodor-Echtermeyer-Weg 1, 14979 Großbeeren, Germany

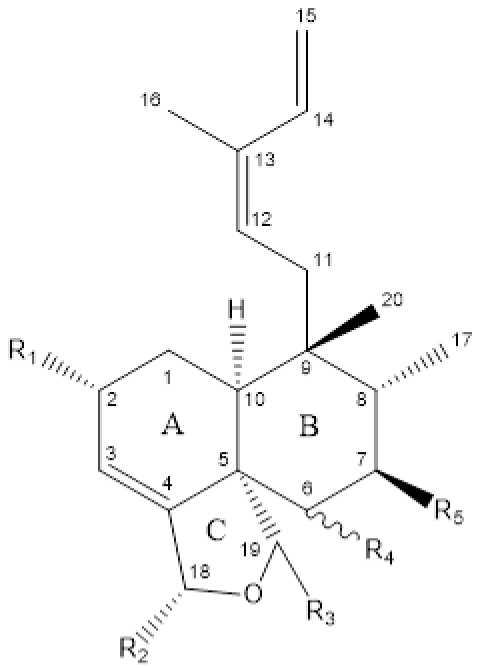

Figure S1: Basic structure of clerodane-type diterpenes isolated from *Casearia* species.

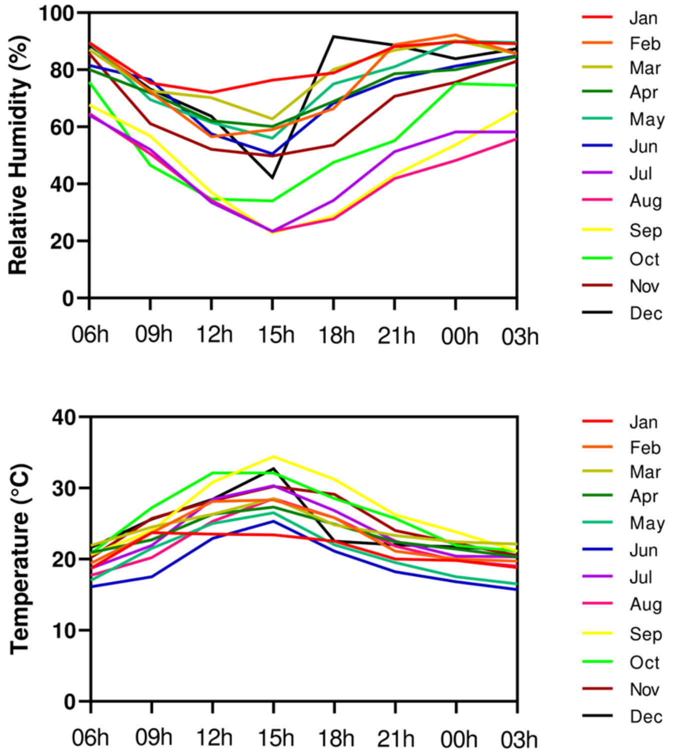

Figure S2. Relative humidity (%) and temperature (°C) monitored at each collection point, during the 12 months of the circadian and seasonal study.

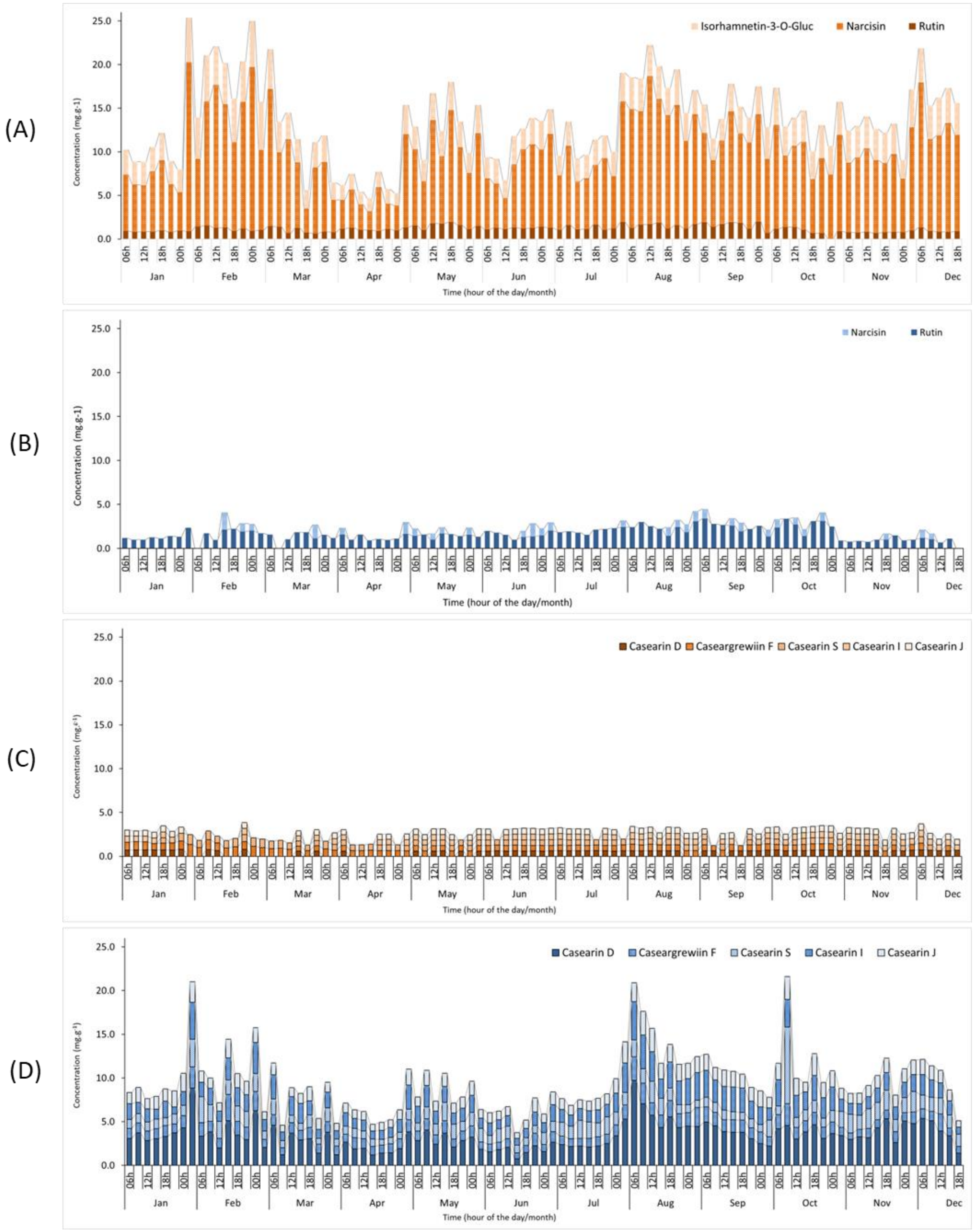

Figure S3. Circadian rhythms of each glycosylated flavonoids and clerodane-type diterpenes in *C. sylvestris var. lingua* (graphs A and C) and *C. sylvestris var. sylvestris* (graphs B and D). The content of each compound was quantified in $mg.g^{-1}$ dry weight using a previously validated chromatographic method.

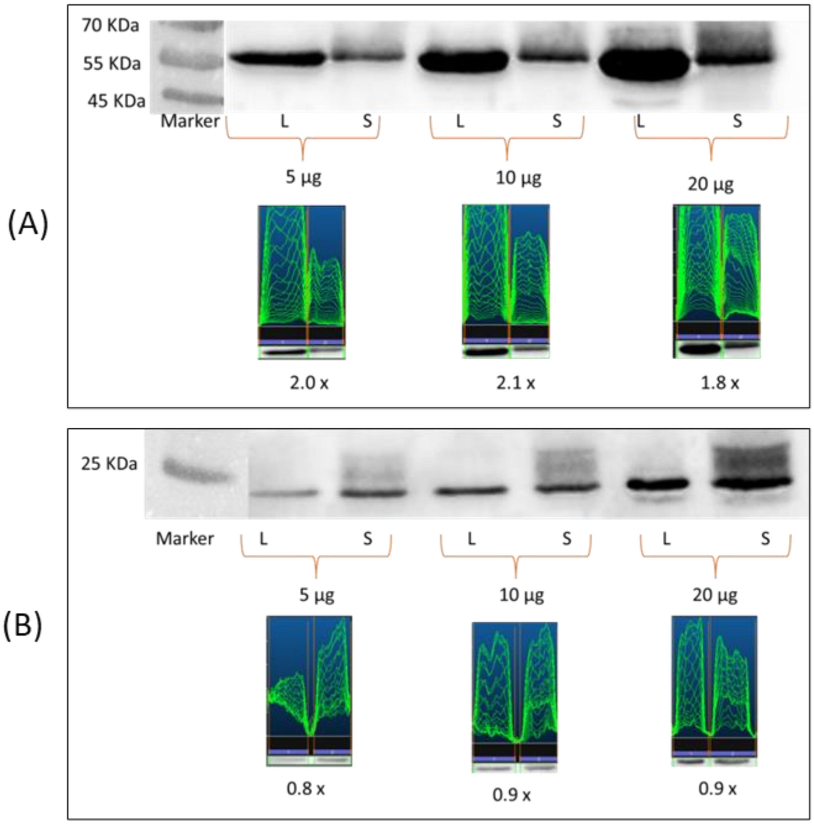

Figure S4. Densitometric analysis of proteins extracted from the two *C. sylvestris* varieties after Western blot. (A) Western blot analysis of the abundance of Rubisco large subunit (55 KDa). (B) Western blot analysis of the abundance of P25K (PSII) protein (25 KDa). The two samples were analyzed in three different concentrations, applying 5 µg, 10 µg and 20 µg. The letter L represents *var. lingua* and the letter S represents *var. sylvestris.*

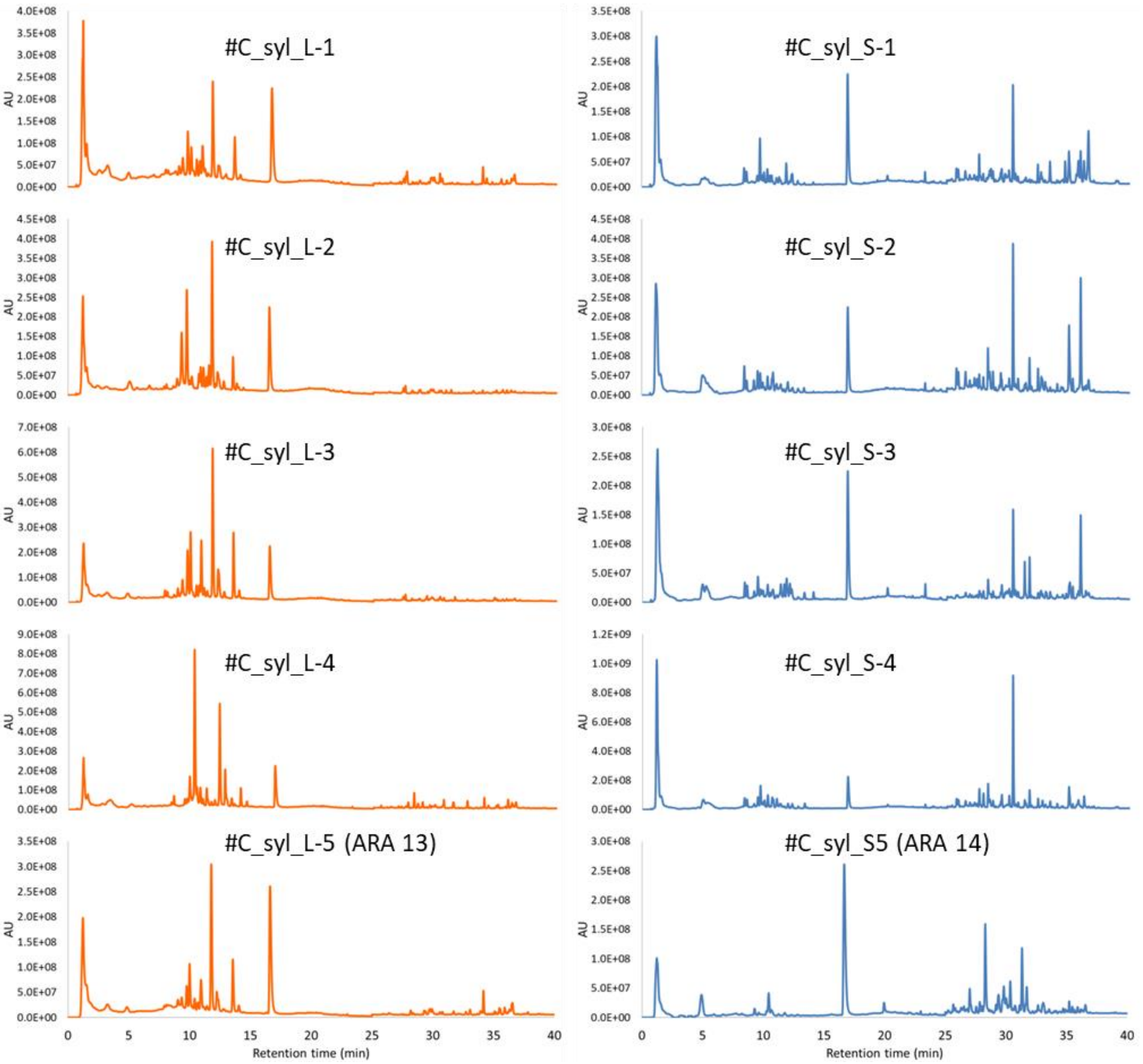

Figure S5. Chromatograms obtained by UHPLC–UV-DAD for the five individuals of *C. sylvestris var. lingua* and (in orange, left) and for the five *C. sylvestris var. sylvestris* (in blue, right), collected in the municipality of Araraquara/SP (Brazil), showing the characteristic chemical profile of each variety. For the circadian and seasonal study, only one individual of each variety was selected (#C_syl_L-ARA 13 and #C_syl_S-ARA 14, also shown in Figure 2 of the main manuscript). Chromatograms were plotted at 254 nm (from 0 to 25 min) and 235 nm (from 25 to 45 min) highlighting the elution regions of glycosylated flavonoids and clerodane-type diterpenes, respectively. Chromatographic conditions: Chromatographic column: Kinetex 150 mm×2.1; 2.6 µm. Mobile phase: water and acetonitrile (ACN), both containing 0.1% of formic acid. Gradient elution: 10–25% of ACN from 0 to 15 min, 25–90% of ACN until 35 min, holding 90% of ACN until 40 min. Flow rate: 400 µL.min^{-1}. Oven temperature: 35 ºC. Injection volume: 2 µL.

Table S1. Comparison between the compound's levels produced by *C. sylvestris* var. *lingua* (L) and *C. sylvestris* var. *sylvestris* (S) in the day-light *versus* night cycles throughout the year. Comparisons were performed using *Mann-Whitney test (sample size = 8; α = 0.05,* considering all measurements made in each day/night period). The time-points 21:00, 00:00, 3:00, and 6:00 were taken as replicates for the night period, and the time-points 9:00, 12:00, 15:00, and 18:00 were taken as replicates for the day-light period.

	Rutin		Narcisin		Isorhamnetin-3-*O*-Gluc		Casearin D		Caseargrewiin F		Casearin S		Casearin I		Casearin J	
	L	S	L	S	L	S	L	S	L	S	L	S	L	S	L	S
Jan	0.3429	0.1143	0.6857	ND	>0.9999	ND	0.6857	0.1143	0.8857	>0.9999	0.1429	0.3429	0.3429	0.6857	0.8857	0.1143
Feb	**0.0286***	0.1143	0.3429	ND	0.4857	ND	>0.9999	0.4857	0.4857	0.4857	0.3429	0.8857	>0.9999	0.1143	>0.9999	0.2000
Mar	0.2000	0.4857	0.3429	ND	0.8857	ND	>0.9999	0.8857	>0.9999	0.8857	>0.9999	0.8857	>0.9999	0.3429	>0.9999	0.6857
Apr	0.1143	0.8857	0.6857	ND	0.8857	ND	>0.9999	0.4857	0.6857	0.8857	0.6857	0.0286	0.1429	0.6857	0.1429	0.8857
May	>0.9999	0.8857	0.4857	ND	0.4857	ND	0.3714	0.8857	0.8857	0.4857	0.6857	>0.9999	0.4857	>0.9999	0.4857	0.6857
Jun	0.3429	0.3429	**0.0286***	ND	0.3429	ND	0.3429	0.6857	0.1143	0.8857	0.4857	0.6857	**0.0286***	0.6857	**0.0286***	>0.9999
Jul	0.6857	**0.0286***	0.3429	ND	0.3429	ND	>0.9999	0.1143	0.4857	**0.0286***	>0.9999	0.4857	0.6857	0.0571	0.6857	0.0571
Aug	0.4857	0.1143	0.4857	ND	0.3429	ND	0.6857	0.2000	0.2000	>0.9999	0.2000	**0.0286***	0.8857	0.4857	0.3429	0.3429
Sep	0.8857	0.2000	0.4857	ND	0.8857	ND	>0.9999	0.1143	0.6857	0.4857	0.8286	0.0571	0.7714	0.8857	0.7714	0.6857
Oct	**0.0286***	>0.9999	0.1143	ND	0.8857	ND	0.3429	0.6857	**0.0286***	0.4857	0.3429	0.6857	0.6857	0.4857	0.6857	0.8857
Nov	0.3429	0.1143	>0.9999	ND	0.3429	ND	>0.9999	0.4857	0.1143	0.2000	**0.0286***	0.6857	0.4857	0.2000	0.4857	0.2000
Dec	0.4857	0.2000	>0.9999	ND	0.6857	ND	0.3429	0.3429	0.7143	0.8857	0.4286	0.8857	0.3429	0.1143	0.3429	0.3429

* p-value < 0.05. ND – not detected

Table S2. Comparison between *C. sylvestris* var. *lingua* and *C. sylvestris* var. *sylvestris* compounds levels throughout the year. Comparisons were performed using multiple t-tests (n=16, considering all measurements made in each month, for each compound), not assuming consistent standard deviation (SD), and using the Holm-Sidak method for adjusting multiple comparisons p-values.

	Rutin	Narcisin	Isorhamnetin-3-*O*-Gluc	Casearin D	Caseargrewiin F	Casearin S	Casearin I	Casearin J
Jan	0.3275	-6243****	-2.84***	2.861***	0.2465**	0.8095***	0.8822****	0.9894****
Feb	0.8957	-14.93****	-4.907***	3.565***	0.7678**	1.234***	2.298****	1.511****
Mar	0.3373	-8566****	-3.425***	2.688***	0.3064**	0.3429	1.586****	1.000****
Apr	0.1341	-3.313****	-1.667***	1.633***	0.3843****	0.1677**	0.8646****	0.6424***
May	0.05423	-8.553****	-2.855***	2.818***	0.5765****	0.7642***	1.351****	0.8686****
Jun	0.2063	-7.11****	-2.805***	1.05***	0.2805**	0.5566**	0.6195	0.493**
Jul	0.7152	-7.194****	-2.693***	1.827***	0.3946****	1.025****	0.9932****	0.7039****
Aug	0.7488	-13.13****	-3.55***	5.189***	1.127****	1.022**	2.643****	1.603****
Sep	0.9489	-10.14****	-2.916***	3.249***	0.8449****	0.6585**	2.18****	1.244****
Oct	1.631	-8.245****	-3.549***	2.948***	0.8037***	0.7032**	1.847****	1.192****
Nov	0.186	-8.498****	-3.437***	3.138***	0.4799**	1.356****	0.8768****	0.7021****
Dec	-0.01219	-12.09****	-3.971***	3.255***	0.9463**	1.44****	1.008****	0.7183****

* p-value < 0.05; ** p-value < 0.01; *** p-value < 0.001; ****p-value < 0.0001
